# No significant difference found in PET/MRI CBF values reconstructed with CT-atlas-based and ZTE MR attenuation correction

**DOI:** 10.1186/s13550-019-0494-9

**Published:** 2019-03-19

**Authors:** Hidehiko Okazawa, Tetsuya Tsujikawa, Yoshifumi Higashino, Ken-Ichiro Kikuta, Tetsuya Mori, Akira Makino, Yasushi Kiyono

**Affiliations:** 1 Biomedical Imaging Research Center, University of Fukui, 23-3, Matsuoka-Shimaizuki, Eiheiji-cho, Fukui 910-1193 Japan; 2 Deartment of Neurosurgery, Faculty of Medical Sciences, University of Fukui, 23-3, Matsuoka-Shimaizuki, Eiheiji-cho, Fukui 910-1193 Japan

**Keywords:** Zero-TE, MR attenuation correction, Quantitative PET/MRI, Cerebral blood flow, IDIF

## Abstract

**Background:**

Accurate attenuation correction (AC) is one of the most important issues to be addressed in quantitative brain PET/MRI imaging. Atlas-based MRI AC (AB-MRAC), one of the representative MRAC methods, has been used to estimate the skull attenuation in brain scans. The zero echo time (ZTE) pulse sequence is also expected to provide a better MRAC estimation in brain PET scans. The difference in quantitative measurements of cerebral blood flow (CBF) using H_2_^15^O-PET/MRI was compared between the two MRAC methods, AB and ZTE.

**Method:**

Twelve patients with cerebrovascular disease (4 males, 43.2 ± 11.7 years) underwent H_2_^15^O-PET/MRI studies with a 3-min PET scan and MRI scans including the ZTE sequence. Eleven of them were also studied under the conditions of baseline and 10 min after acetazolamide administration, and 2 of them were followed up after several months interval. A total of 25 PET images were reconstructed as dynamic data using 2 sets of reconstruction parameters to obtain the image-derived input function (IDIF), the time-activity curves of the major cerebral artery extracted from images, and CBF images. The CBF images from AB- and ZTE-MRAC were then compared for global and regional differences.

**Results:**

The mean differences of IDIF curves at each point obtained from AB- and ZTE-MRAC dynamic data were less than 5%, and the differences in time-activity curves were very small. The means of CBF from AB- and ZTE-MRAC reconstructions calculated using each IDIF showed differences of less than 5% for all cortical regions. CBF images from AB-MRAC tended to show greater values in the parietal region and smaller values in the skull base region.

**Conclusion:**

The CBF images from AB- and ZTE-MRAC reconstruction showed no significant differences in regional values, although the parietal region tended to show greater values in AB-MRAC reconstruction. Quantitative values in the skull base region were very close, and almost the same IDIFs were obtained.

## Introduction

A new hybrid scanner combining positron emission tomography (PET) and MRI, the PET/MRI system, should be very beneficial in the neuropsychiatric field, especially for the precise fusion of high-grade anatomical MR images and PET molecular imaging [1]. The quantitative PET images are also anticipated, although several issues remain to be confirmed for global as well as regional values. MR-based attenuation correction (MRAC) is one of the major concerns in quantitative brain PET images because the brain is covered by the skull. MR segmentation images using the Dixon method are usually applied for the reconstruction of body PET images. However, for brain PET images, MRAC using the Dixon-based method showed greater bias and underestimation of PET values compared with CT-based attenuation correction methods because of inaccurate estimation of the bone attenuation [2, 3]. The single- and multi-CT atlas-based (AB) attenuation correction methods were also compared, and multi-AB-MRAC showed PET quantitative values very close to those from individual CT attenuation correction (CTAC) [4]. The ultrashort echo time (UTE) and zero echo time (ZTE) pulse sequences have been applied to brain PET image reconstruction for bone identification in the MRAC method, and biases of quantitative values were reported to be reduced [5–7]. A multicenter study showed that these newer MRAC methods produced better and more reliable quantitative images than those from the simple Dixon-based method [8]. These recent studies suggested that the MRAC method has been improved in terms of quantitative PET evaluation using PET/MRI scanners.

PET measurement of cerebral blood flow (CBF) using H_2_^15^O PET usually requires arterial blood sampling from individual patients to estimate the input function of the tracer. Recently, several methods to obtain the image-derived arterial input function (IDIF) have been proposed by using time-of-flight (TOF) MR angiography images to detect the location of the internal carotid arteries (ICA) at the skull base [9–12]. The method is ideal for the PET/MRI scanner because both image data can be acquired simultaneously. The IDIF method in H_2_^15^O PET/MRI is useful and practical to estimate the arterial input function directly from the cerebral arteries [13], and the CBF values using IDIF were consistent with previous ^15^O-PET studies [14, 15]. However, the brain counts in the skullbase were reported to be underestimated in previous studies [4, 6], while a recent study suggested that both AB- and ZTE-MRAC tended to overaetimate PET counts slightly in the whole brain even in the skullbase [16]. The PET counts at the skullbase may influence on the quantitative values in the IDIF method, because it usually uses arterial counts at the ICA in the skullbase area.

The present study investigated the differences of IDIF curves at the ICA area using the two different MRAC methods as well as their influence on CBF values in the whole brain. Since the ZTE approach is reported to show closer PET counts to ordinary CTAC, AB-MRAC may provide greater bias in brain PET images and IDIF counts [16].

## Material and methods

### Subjects

Twelve patients with cerebrovascular disease (4 males and 8 females, 43.2 ± 11.7 years) were studied using H_2_^15^O PET/MRI for measurement of CBF. All patients had unilateral stenosis or occlusions in the internal carotid artery (ICA) or the middle cerebral artery (MCA). Eleven of them underwent H_2_^15^O PET/MRI scans before and after the acetazolamide injection to evaluate their hemodynamic status before considering further treatment [17]. One underwent only baseline CBF evaluation, and two were followed up to observe hemodynamic changes after the neurosurgical treatment (carotid artery stenting). Thus, 25 H_2_^15^O PET/MRI scans were performed in total. The study was approved by the Ethics Committee of the University of Fukui, Faculty of Medical Sciences, based on its guidelines (Ethical Guidelines for Medical Science Research with Humans) as well as the Helsinki Declaration of 1975 (revised in 1983). Written informed consent was obtained from each patient.

### PET/MRI scanner preparation

A whole-body PET/MRI scanner (Signa PET/MR, ver. 24, GE Healthcare, Milwaukee, WI, USA) was used for simultaneous PET and MRI data acquisition [18]. The scanner permits PET acquisition of 89 image slices in three-dimensional (3D) mode with a slice thickness of 2.45 mm. Performance tests showed the intrinsic resolution of PET images to be 4.2–4 .3 mm in the transaxial direction. The PET/MRI scanner was calibrated with a dose-calibrator (CRC-12, Capintec Inc., NJ, USA) beforehand using a pool phantom and ^18^F-solution, according to the manufacturer’s scanner guidelines.

### PET and MRI data acquisition

The patients underwent brain PET/MRI scans using a standard head coil (8-channnel HD Brain, GE Healthcare) for simultaneous PET and MRI acquisition. A 3-min list-mode 3D PET scan in TOF acquisition mode was started at the time of a bolus tracer injection of 555 MBq ^15^O-water via the antecubital vein. During the PET scan, LAVA-Flex (GE Healthcare) T1-weighted images (repetition time, 4 ms; echo time, 1 .7 ms; flip angle, 5°; slice thickness, 5 .2 mm with 2 .6 mm overlap; 120 slices; pixel size, 1.95 × 2.93 mm; acquisition time, 18 s) were acquired for MRAC in the head position using the default CT atlas-based map [19]. A 3D radial acquisition was also performed for the ZTE approach in the axial direction with the following parameters: field of view (FOV) 264 mm, matrix 110 × 110 × 116, voxel size 2.4 × 2.4 × 2.4 mm^3^, flip angle 0.8°, number of excitations 4, bandwidth ± 62.5 kHz, and acquisition time of 41 s [20, 21]. MR angiography (MRA) and other anatomical MR images such as 3D-T1WI, T2WI, fluid-attenuated inversion recovery (FLAIR), etc. were acquired in the same position after the PET scan.

### AB- and ZTE-based attenuation map

The CT atlas-based attenuation correction (AC) map (AB-MRAC) was calculated from the LAVA-Flex images using the system’s default processing [19]. The head volume was rescaled automatically to annihilate photon attenuation coefficients [22]. The following process was performed on a research tool platform (PET tool box, GE Healthcare) to create the ZTE-MRAC map based on the previous study [20]. Briefly, the first step was pre-filtering and histogram-based normalization, followed by an intensity-based segmentation of the head, bias correction, identification of voxels affected by partial volume effects, and segmentation of the sinus, bone, and cavity masks. After these steps, a pseudo-CT map was generated with bone tissue linearly scaled based on the ZTE intensity. This mapping was determined by fitting of registered CT and ZTE data in the bone density range. To convert the pseudo-CT into a MRAC map, the images were re-sampled with a 60 × 60 × 25 cm^3^ FOV in 128 × 128 × 89 matrix, and finally re-scaled to 511 keV attenuation coefficients.

### PET reconstruction and CBF image calculation

The dynamic PET images were reconstructed from the PET data using the 3D ordered-subset expectation maximization (OSEM) method and point spread function (PSF) modeling algorithm in 21 frames of 12 × 5 s, 6 × 10 s, and 3 × 20 s. Two OSEM parameter sets were applied for PET image reconstruction as follows: (A) subset, 32; iteration, 4; transaxial post-reconstruction Gaussian filter cutoff, 2 mm; and (B) subset, 28; iteration, 3; Gaussian filter cutoff, 3 mm in 256 mm FOV and 2 × 2 mm pixel size. Image data set A was used for estimation of the arterial input function using the IDIF method, and image data set B was used for CBF image calculation [13]. Decay of radioactivity in the dynamic PET data was corrected to the starting point of each scan.

Details of the IDIF extraction are described elsewhere [13]. In brief, the average image of the initial phase (10–40s) of dynamic PET data was used to extract voxels only inside the ICA, where the 30 most intensely radioactive voxels were selected as the volume of interest (VOI) mask at the region of the cavernous part of the ICA. Individual 3D TOF-MRA images were used to confirm the position of the arterial VOI mask exactly on the ICA [12, 13]. Time-activity curves (TACs) for IDIF were obtained by applying the ICA VOI masks on the dynamic PET data.

CBF images (mL/min/100 g) were calculated by the autoradiographic method with individual look-up tables using arterial IDIF and a partition coefficient of 0.9 [13, 15, 23]. Since the IDIF was obtained from the same dynamic PET data at the skull base, no correction for the delay time of tracer arrival was needed.

### Statistical analyses in CBF values

**Fig. 1:**
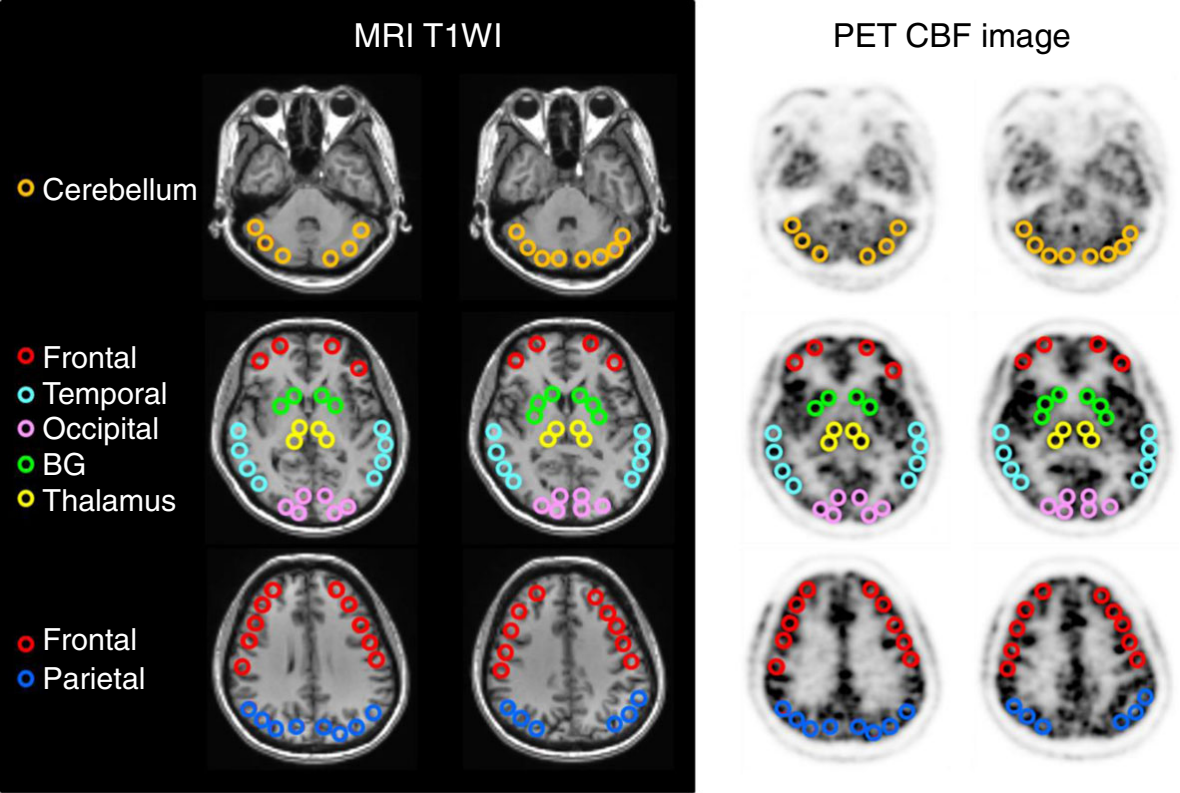
ROIs on several representative slices of MRI (left two columns) and PET (right two columns). Different colors shows different locations of frontal (red), temporal (cyan), occipital (light pink), parietal (blue) lobes, basal ganglia (green), thalamus (yellow), and cerebellum (orange). ROIs were placed in the MRI images and transferred to the CBF-PET in the same location

To compare the cortical CBF values between the two MRAC methods, 10–30 circular regions of interest (ROIs) 10-mm in diameter were set manually for each region of the bilateral hemispheres using the individual 3D-T1WI images (Fig. 1). Dr. View (AJS Co., Ltd., Tokyo, Japan) was used for the ROI analysis. Large regions such as the cerebral cortices and the cerebellum include 25–30 ROIs for each hemisphere and small brain regions such as the basal ganglia and the thalamus includes 10 ROIs. CBF values in the affected region and infarction, if any, were excluded. Each regional value was the average of ROIs in the same region. Two of the authors who are radiologists (HO and TT) determined the location of the ROIs and confirmed they were appropriate. Repeated measures analysis of variance (ANOVA) with a post-hoc paired *t* test was applied to analyze the difference in the mean cortical CBF values between the two MRAC methods of AB and ZTE. *P* < 0.05 was considered to be statistically significant. To observe regional correlation of the two methods, CBF values from 310 ROIs in total were plotted for each patient. Bland-Altman plot analysis was also applied to the result when a wide variation was observed in the scatter plot.

## Results

**Fig. 2:**
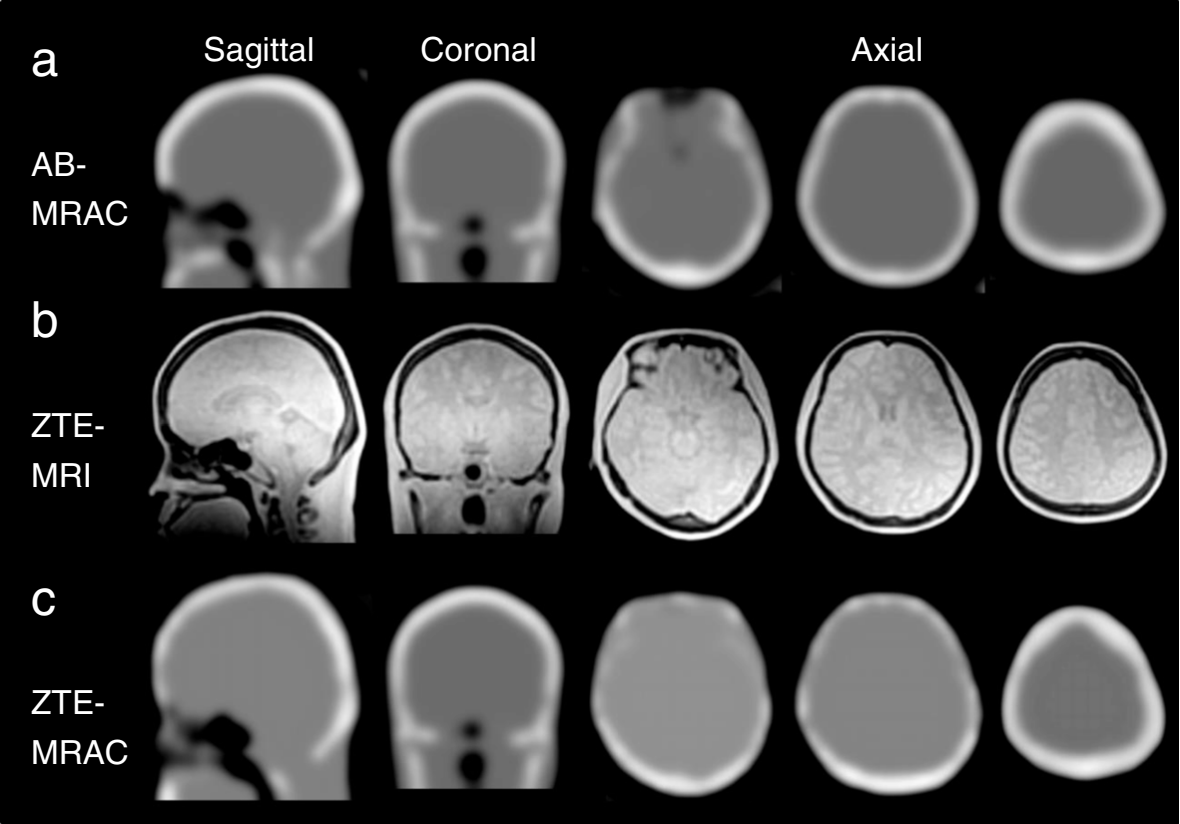
MRAC maps from the AB- (**a**) and ZTE-based (**c**) methods, and ZTE images (**b**) for PET attenuation correction. Although the ZTE-MRAC shows similar head AC maps to AB-MRAC, there are slight differences in the frontal sinus region (left column of axial sections) and bone thickness in the parietal region (middle and right column of axial sections)

Figure 2 shows MRAC maps and ZTE images for the PET attenuation correction. Although the ZTE-MRAC (Fig. 2c) shows similar head AC maps to AB-MRAC (Fig. 2a), slight differences can be observed in the frontal sinus region and thickness of skull bone, especially in the frontal part.

**Fig. 3:**
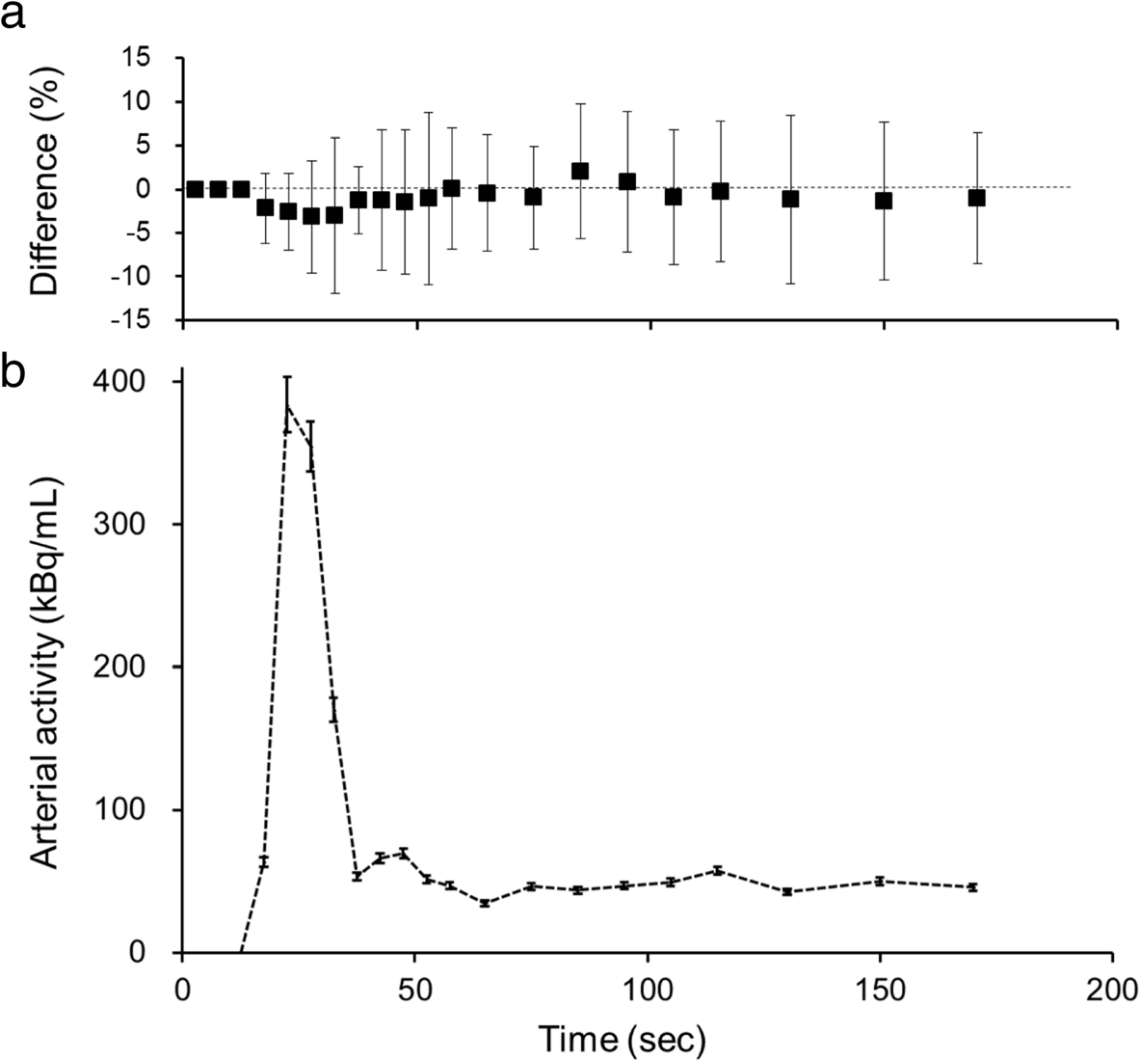
**a** Average percentage differences in arterial radioactivity at each point of dynamic PET frames. The difference of each point was less than 5%, although variance (SD) was about ± 10%. **b** A representative IDIF curve from a patient is presented at the bottom. The error bars were calculated from the average difference of each time point. Estimation errors are small, especially in the phase of low radioactivity concentration

**Fig. 4:**
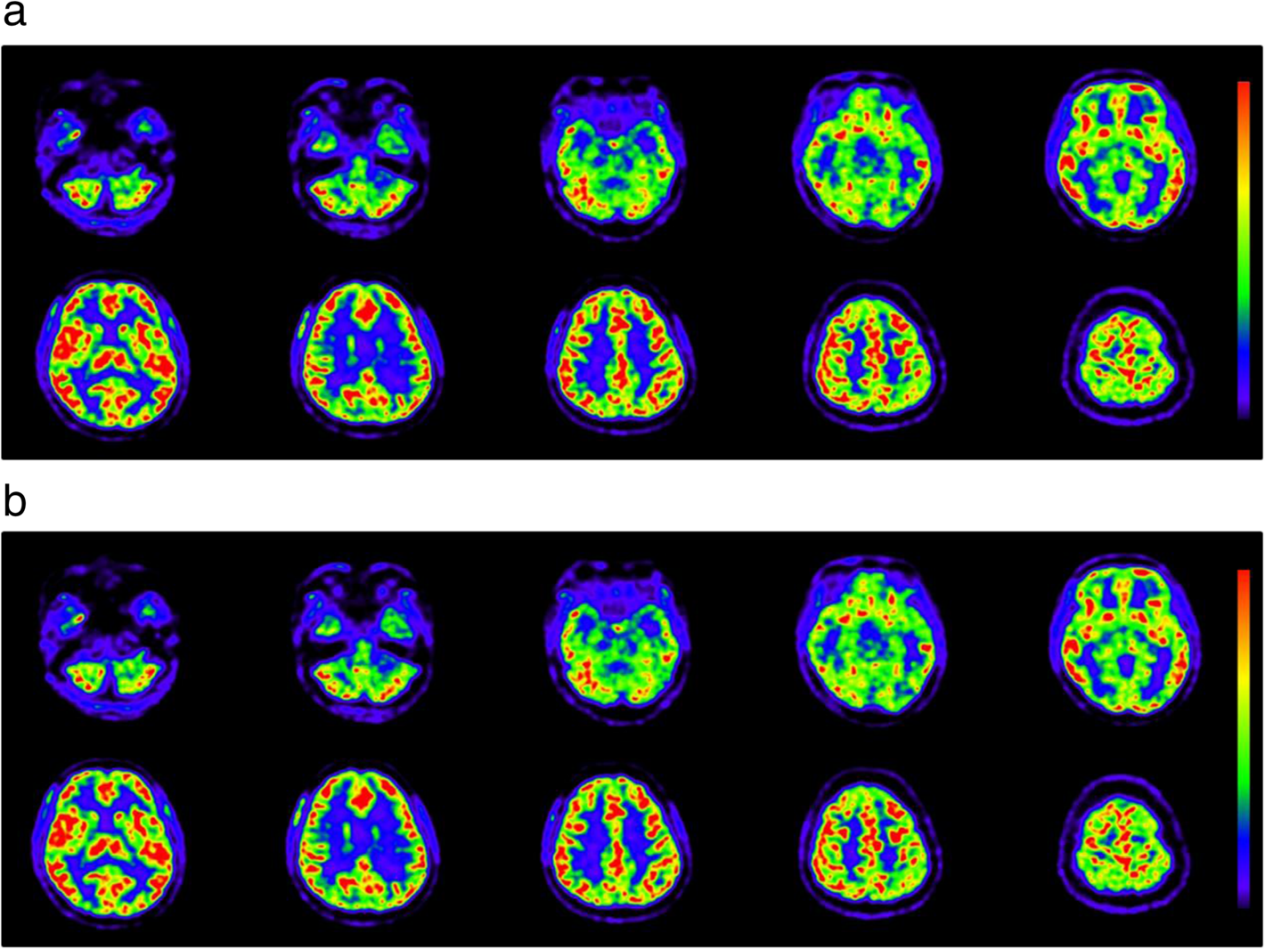
Representative CBF images calculated from dynamic PET data reconstructed by the AB- (**a**) and ZTE-MRAC (**b**) methods. Color scales in the same range show that the two images are very close and regional values are almost the same

**Fig. 5:**
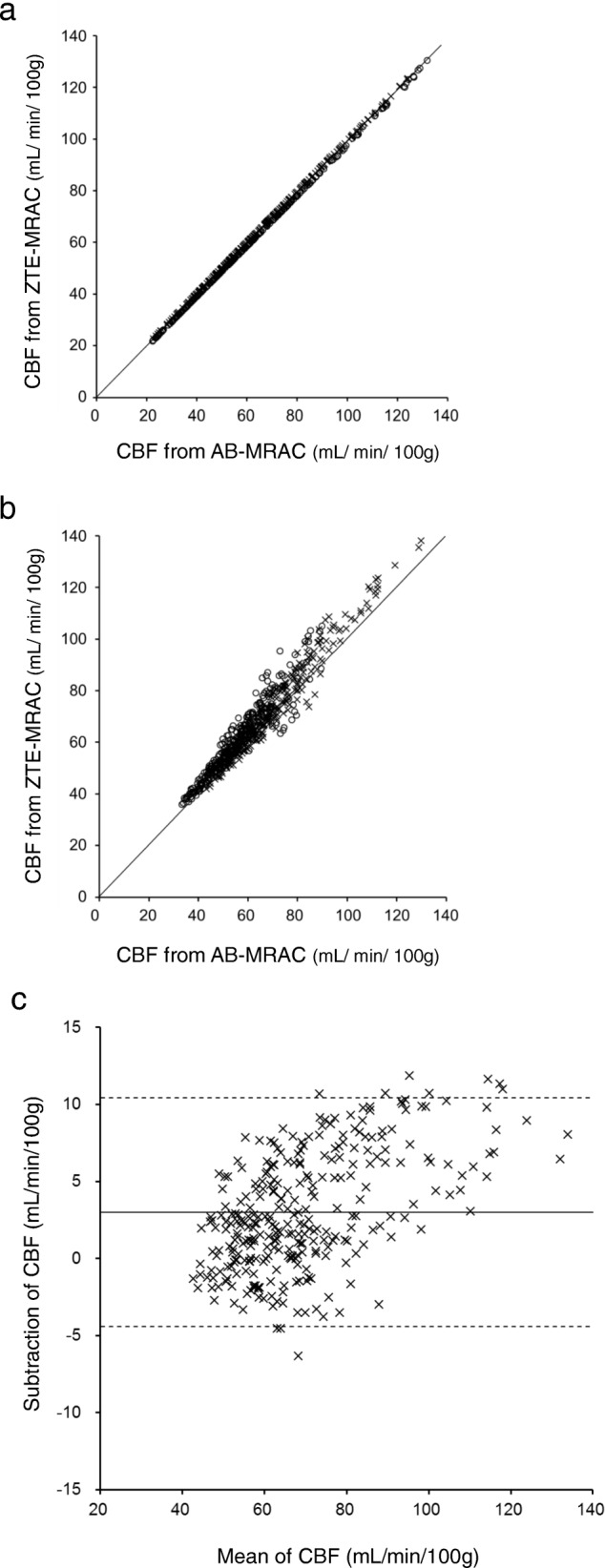
Two representative regression analyses for regional CBF obtained from 310 ROIs for all brain regions (see Table 1). The first case (**a**) showed excellent correlation at baseline (○: *y* = 0.99*x* + 0.48, *R*^2^ = 1.0) as well as after acetazolamide administration (×: *y* = 0.99*x* + 0.13, *R*^2^ = 1.0). Several cases showed slightly greater variance, as shown in panel (**b**), although the correlation coefficient was excellent (baseline (○): *y* = 1.1*x* + 4.5, *R*^2^ = 0.96; acetazolamide (×): *y* = 1.1*x* + 1.3, *R*^2^ = 0.90). Graph **c** shows the Bland-Altman plot for the second case which had no significant bias, although the AB-MRAC CBF image tended to show greater values in the higher range of the CBF mean (mean difference 3.0 ± 3.8; range − 4.4–10.4 mL/min/100 g)

Figure 3 shows the average differences in arterial radioactivity at each point of the dynamic PET frame. The differences were less than 5%, although the variances (SD) were as large as ± 10% (Fig. 3a). Figure 3b shows a representative IDIF curve from a patient with error bars calculated from the average differences of each time point. Estimation errors were small, especially in the phase of lower radioactivity, indicating that only small differences may be expected in the area under the curve of IDIF from the AB- and ZTE-MRAC PET images. As the result of this small difference in IDIF curves, the CBF images calculated from AB-MRAC reconstruction and those from ZTE-MRAC were very close, and the regional values were almost the same (Fig. 4). Table 1 shows the regional average values at baseline and after the acetazolamide challenge test. The regional values averaged for the bilateral hemispheres showed no significant differences between the two MRAC methods. A scatter plot of the regional CBF values from the 310 ROIs used for CBF measurement showed a good correlation. Figure 5 shows two representative cases of regression analysis; one of them had excellent correlations for both at baseline and after acetazolamide administration (Fig. 5a, *R*^2^ = 1.0). Even in the second case, which had greater variance, the correlation coefficient was excellent (Fig. 5b, *R*^2^ = 0.96). The means of slopes and correlation coefficients for all PET scans were 1.02 ± 0.08 and 0.97 ± 0.03, respectively. The Bland-Altman plot for the latter case showed no significant bias, although the CBF image with AB-MRAC tended to show greater values in the higher mean CBF range (mean difference 3.0 ± 3.8; range − 4.4–10.4 mL/min/100 g) (Fig. 5c).

**Table 1:** Regional CBF values (mL/min/100 g) from two MRAC methods (mean ± SD)

	AB baseline (*n* = 14)	ZTE baseline (*n* = 14)	AB ACZ (*n* = 11)	ZTE ACZ (*n* = 11)
Frontal	46.7 ± 14.0	45.2 ± 12.5	50.9 ± 15.2	50.6 ± 15.0
Parietal	45.6 ± 10.7	44.1 ± 9.7	56.4 ± 13.0	56.0 ± 12.2
Temporal	45.0 ± 11.9	45.6 ± 11.6	59.5 ± 15.4	60.4 ± 15.6
Occipital	45.6 ± 12.4	45.4 ± 12.3	64.6 ± 21.2	64.6 ± 20.8
Basal ganglia	50.9 ± 13.9	51.4 ± 13.5	60.9 ± 13.5	61.3 ± 13.5
Thalamus	53.3 ± 16.9	53.6 ± 16.6	78.7 ± 25.1	79.3 ± 24.6
Cerebellum	51.1 ± 13.2	52.2 ± 13.4	68.9 ± 18.5	70.1 ± 19.6

*AB* CT atlas-based method, *ZTE* zero echo time MRI-based method, *ACZ* acetazolamide

**Fig. 6:**
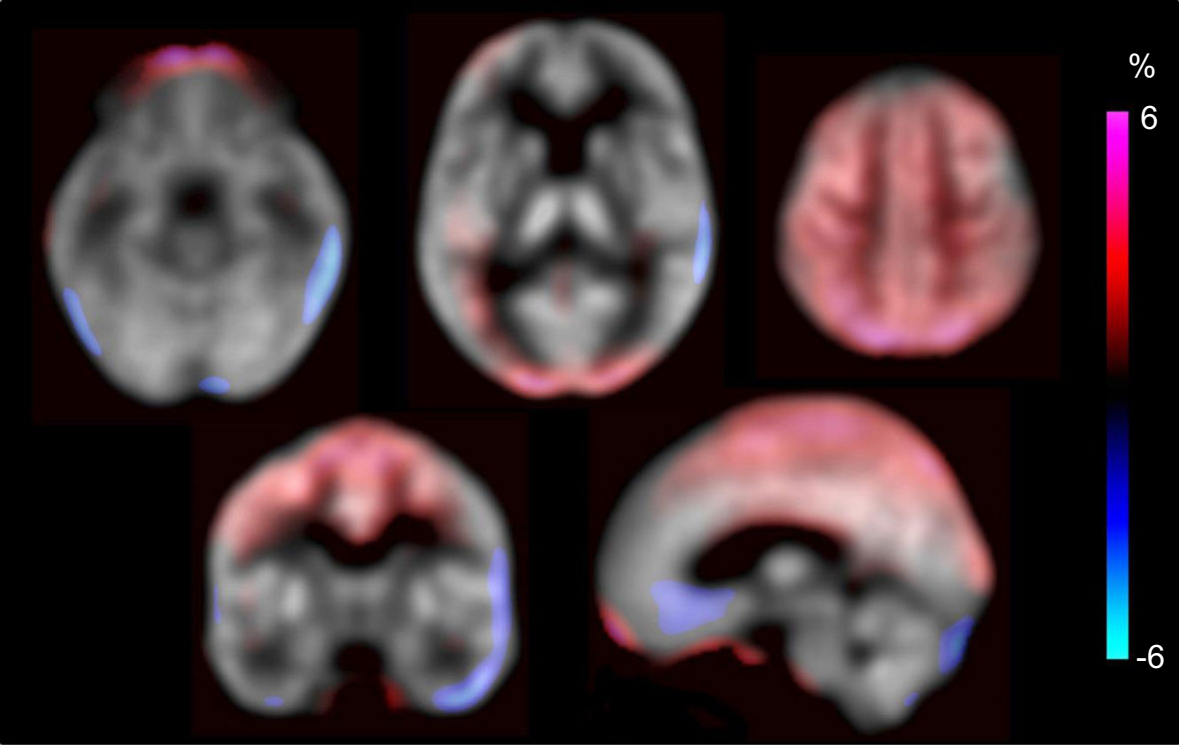
Average image of percentage differences between AB- and ZTE-MRAC CBF calculated from all individual percentage change images. Red and blue indicate higher and lower values in the AB-MRAC method, compared with the ZTE-MRAC method, respectively. The parietal to occipital region shows higher CBF values with the AB-MRAC method compared with the ZTE-MRAC method, although the difference was less than 5%. Most of other brain regions showed very close regional CBF values, less than ± 2%

Figure 6 shows the mean percentage differences in CBF images calculated from individual CBF by 2 × [AB-MRAC − ZTE-MRAC] / [AB-MRAC + ZTE-MRAC]. Red color indicates higher values and blue indicates lower values for the AB-MRAC method. The parietal to occipital region shows higher CBF values for the AB-MRAC method compared with those of the ZTE-MRAC method, although the difference was less than 5%. Other brain regions showed no differences and the quantitative CBF values were very close.

## Discussion

The present study evaluated the effects of a new MRAC method using ZTE images on the quantitative PET and CBF values calculated from brain H_2_^15^O PET/MRI. We compared arterial input curves from the IDIF method and regional CBF values calculated separately from two different dynamic PET images of AB- and ZTE-MRAC reconstruction, and found no significant differences between the two methods. The TACs from the IDIF showed mean differences less than 5% at each time point, and did not show significant differences in the areas under the curves of the TACs. The 3-min H_2_^15^O-PET average images as well as quantitative CBF images from the AB-MRAC reconstruction method tended to show greater regional values compared with the ZTE-MRAC reconstruction method (Fig. 6). However, the values at the skull base, where the IDIF-TAC was obtained, were similar. Previous FDG-PET studies also showed a tendency of overestimation of AB-MRAC images in the parietal region compared with images from CTAC [6, 21]. Thus, the ZTE-MRAC method seems to be appropriate for the quantitative evaluation of brain PET. Our previous study showed that overall CBF values from H_2_^15^O PET/MRI using the IDIF method were reasonable in healthy volunteers [13], and were consistent with a previous multicenter study in Japan using ^15^O-PET for healthy volunteers [14].

There were no significant differences in the regional CBF values from each brain area obtained from patients with cerebrovascular disease in the present study. Although recent FDG-PET/MRI studies showed little regional differences in PET counts when using several new MRAC methods [5, 21], about 5% regional difference at maximum may affect results of statistical mapping analysis if we use normal database obtained from different calculation methods of PET image. To apply brain PET/MRI images for statistical mapping analysis, the same method of PET/MRI reconstruction should be used for both control as well as patient images.

Several previous studies reported that the AB-MRAC method provided reasonable PET values, although AB-MRAC tended to overestimate in the parietal regions, and the ZTE-MRAC method showed a tendency of slight underestimation for the whole brain [4, 6, 21]. These results, comparing FDG accumulation in the brain, are consistent with the present study. If we had compared our CBF data with images from other AC methods such as CTAC, similar results may have been observed because the ZTE-MRAC method would provide quantitative images very close to the CTAC PET images [16]. One of the reasons for the higher CBF values in the parietal region with the AB-MRAC method may have been caused by the thickness of the skull CT atlas prepared by the vendor. The frontotemporal part of the upper skull showed a thicker bone map on AB-MRAC images compared with ZTE-MRAC (Fig. 2, right column in axial view). This small difference may have induced the overestimation in AB-MRAC images.

**Fig. 7:**
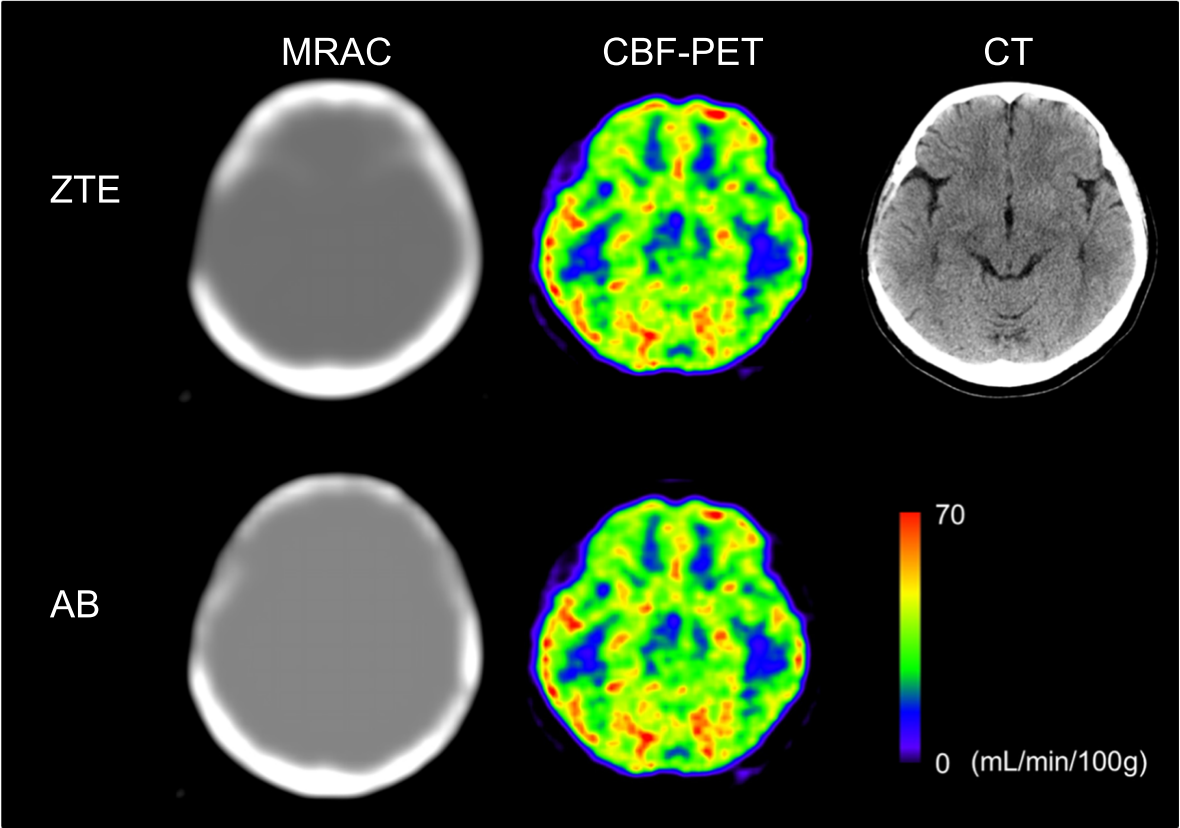
ZTA-, AB-MRAC, and CBF images at the same slice level from a patient who underwent neurosurgical treatment. Top row shows the ZTE-MRAC, CBF, and CT at the corresponding slice level (right). The defect of the right temporal bone is clearly delineated by the ZTE-MRAC (left). The bottom row shows the images from the AB-MRAC method (left), which generated the right temporal bone. CBF images from the two methods show that CBF values in the right temporal lobe were very close (middle column). This PET scan was not included in the analysis

The TOF technique for PET reconstruction using PET/MRI seems to be important, especially for quantitative images. The frontal part of the skull sometimes shows substantial differences between AB-MRAC and CTAC due to the frontal sinus. If the difference between MRAC and CTAC strictly affects the PET image calculations, brain cortices close to the sinus would show significant differences in regional radioactivity concentration, as well as on quantitative images. However, even in the cases with a relatively large frontal sinus as well as temporal bone defects after neurosurgical operation, the CBF values close to the bone defect were not significantly different (Fig. 7). This result indicates that the TOF acquisition technique would substantially compensate for the errors in MRAC [6, 24]. TOF reconstruction is reported to be less sensitive to errors in attenuation correction, and is beneficial in multimodal systems such as PET/MRI [25].

A limitation of the present study was that we evaluated only a small number of patients with cerebrovascular disease who had unilateral occlusive changes in the major cerebral arteries. Since each IDIF was obtained from an unoccluded part of the internal carotid artery, and all patients had excellent input curves very close to those of healthy volunteers [13], the IDIF curves were considered to be appropriate and did not affect the CBF calculation. Furthermore, the purpose of the study was to compare the difference in quantitative results obtained from two different MRAC methods. Although the patient number was limited, 25 PET scans were compared in total. Thus, we consider that the results of the present study are reliable.

## Conclusion

The CBF images calculated from AB- and ZTE-MRAC reconstruction showed no significant differences in quantitative regional values, although the parietal region tended to show greater values on AB-MRAC images compared with ZTE-MRAC. Arterial radioactivity at the skull base was very similar between them, and almost the same IDIFs were obtained, with small errors, which provided similar CBF values for the brain. Since the commercially available single-CT-atlas-based method transforms the CT-atlas into AB-MRAC using individual Dixon-MRI images, it may induce slight bias on MRAC maps as well as regional PET counts [4, 6, 16]. The ZTE-MRAC reconstruction method, estimating bone attenuation from individual ZTE-MRI images, is expected to be used as a standard method for quantitative brain PET studies.

## Abbreviations

3D: Three dimensional
AB: CT atlas-based
AC: Attenuation correction
ANOVA: Analysis of variance
CBF: Cerebral blood flow
CTAC: CT attenuation correction
FLAIR: Fluid-attenuated inversion recovery
FOV: Field of view
ICA: Internal carotid artery
IDIF: Image-derived input function
MCA: Middle cerebral artery
MRA: MR angiography
MRAC: MR attenuation correction
OSEM: Ordered subset expectation maximization
PSF: Point spread function
ROI: Region of interest
TAC: Time-activity curve
TOF: Time of flight
UTE: Ultra-short TE
VOI: Volume of interest
ZTE: Zero-TE
